# Navigating a Quandary in Kidney Exchange Programs: A Review of Donor Travel versus Organ Shipment

**DOI:** 10.3389/ti.2025.14804

**Published:** 2025-11-12

**Authors:** Mattheüs F. Klaassen, Marry de Klerk, Frank J. M. F. Dor, Sebastiaan Heidt, Stijn C. van de Laar, Robert C. Minnee, Jacqueline van de Wetering, Liset H. M. Pengel, Annelies E. de Weerd

**Affiliations:** 1 Department of Internal Medicine, Erasmus MC Transplant Institute, University Medical Center Rotterdam, Rotterdam, Netherlands; 2 Division of HPB and Transplant Surgery, Department of Surgery, Erasmus MC Transplant Institute, Erasmus Medical Center, Rotterdam, Netherlands; 3 Department of Surgery and Cancer, Imperial College, London, United Kingdom; 4 Erasmus MC Transplant Institute, University Medical Center Rotterdam, Rotterdam, Netherlands

**Keywords:** organ shipment, donor travel, kidney paired donation, kidney transplantation, living donor

## Abstract

In multicenter kidney exchange programs (KEPs), either the explanted kidney must be shipped, or the donor must travel to the transplanting center. This review describes the available data on these two approaches and formulates recommendations for practice. We searched for studies addressing organ shipment or donor travel in KEPs. Data were categorized into four domains: cold ischemia time (CIT), logistics, donor/recipient perspectives and professional perspectives. From 547 articles screened, 105 were included. Kidneys are shipped in most countries. Prolonged CIT due to shipment may increase the risk of delayed graft function, but does not seem to impact graft survival. Planning the shipment requires a robust logistical framework with guaranteed operating room availability. Donor travel is reported to be both emotionally and financially distressing for donors and exposes them to inconsistencies in donor evaluation and counseling across centers. Reduced willingness to participate in KEP when travelling was reported by 36%–51% of donors. Professionals generally support offering organ shipment to donors not willing to travel. In conclusion, the decision between donor travel or organ shipment should be tailored to local circumstances. Healthcare professionals should prioritize minimizing barriers to KEP participation, either by facilitating organ shipment or reducing the burden of donor travel.

## Introduction

Living donor kidney transplantation is the optimal treatment for end-stage kidney disease [1, 2]. While desensitization enables incompatible kidney transplantation, it comes with a higher immunosuppressive burden and inferior outcomes [3–5]. Kidney exchange programs (KEPs) provide a viable alternative, allowing recipients to receive a blood-type or Human Leukocyte Antigen (HLA) compatible kidney by making alternative donor-recipient combinations through exchange chains [6, 7].

The success of KEPs depends on the size and HLA diversity of the donor pool [8–10], particularly for highly immunized patients that are currently accumulating in KEPs [11]. Nevertheless, multicenter KEPs can be challenging; matched donors and recipients are often located in distant transplant centers. To overcome this, the donor must travel to the transplanting center, or the kidney must be shipped between centers after procurement in the donor hospital [12]. Recipient surgeries are typically performed at the initial evaluating center, as this safeguards continuous care for the recipient and these patients face travel limitations due to their kidney disease [13–16]. In contrast, donors are generally healthy and therefore expected to be able to travel.

Shipping donor kidneys will likely increase cold ischemia time, potentially affecting graft outcomes [17, 18]. In addition, donor nephrectomy and kidney implantation are performed in different centers, requiring transplant professionals to cooperate and arrange logistics for transport [19]. Donor travel, while logistically simpler, places a greater burden on donors and might create a disincentive for KEP participation [20–22].

The geographical separation of transplant centers poses a dilemma for multicenter KEPs [12, 23–26]: the travel burden could reduce donor participation, while organ shipment introduces medical, logistical, and financial complexities. A review of pros and cons of both modalities is currently lacking. We aim to provide an overview of this dilemma by analyzing the available data on cold ischemia time (CIT), logistics, donor/recipient perspectives and professional perspectives.

## Methods

We performed a systematic search and review [27]. This entails that we did perform a systematic search to identify all the relevant studies. Since the relevant data were often not the primary topic of included studies, it was not deemed appropriate to perform a formal quality and risk of bias assessment. We narratively synthesized the included data and summarized study data in tables. Based on the synthetized data, recommendations were formulated for clinical practice.

### Literature Search

We conducted a systematic search of multiple databases up to December 20, 2024. The search strategy incorporated terms for living donor kidney transplantation, kidney exchange, organ shipment and donor travel (Supplementary Table S1).

### Inclusion and Exclusion Criteria

Studies describing data on pros and cons of organ shipment or donor travel in KEP were included. Articles not published in English and conference abstracts were excluded. We excluded studies not specifically addressing KEP donors or unspecified donors (UDs), except for studies on CIT for which we also included articles describing living donor transplants in general.

### Additional Data Collection

To provide context with current KEP practices worldwide, we searched the literature and Internet on the policy (donor travel, organ shipment, or combined) and transplant volume (annual KEP transplants and total living donor kidney transplants) of countries with multicenter KEPs. In case of missing data, we contacted KEP representatives via e-mail.

### Screening

Two reviewers (MtK, MrK) independently screened the articles based on title/abstract and full text subsequently. Citation searching of the included studies was performed to find additional, relevant articles. Discrepancies were discussed between the two reviewers. If no consensus was reached, a third reviewer (AW) provided the final decision.

### Data Extraction

For each of the four domains, i.e., CIT, logistics, donor/recipient perspectives and professional perspectives, the first author (MtK) grouped the studies and extracted the relevant data. This included study characteristics (study type, year of publication, number and type of participants, and country) and any data on the pros and cons of organ shipment or donor travel. Extracted data were validated by the second author (MrK).

### Data Analysis

A narrative synthesis of the included studies was performed, and study data were summarized in tables. To avoid the inclusion of duplicate study data, we identified overlapping cohorts and presented the data accordingly in the tables.

## Results

### Inclusion

**FIGURE 1:**
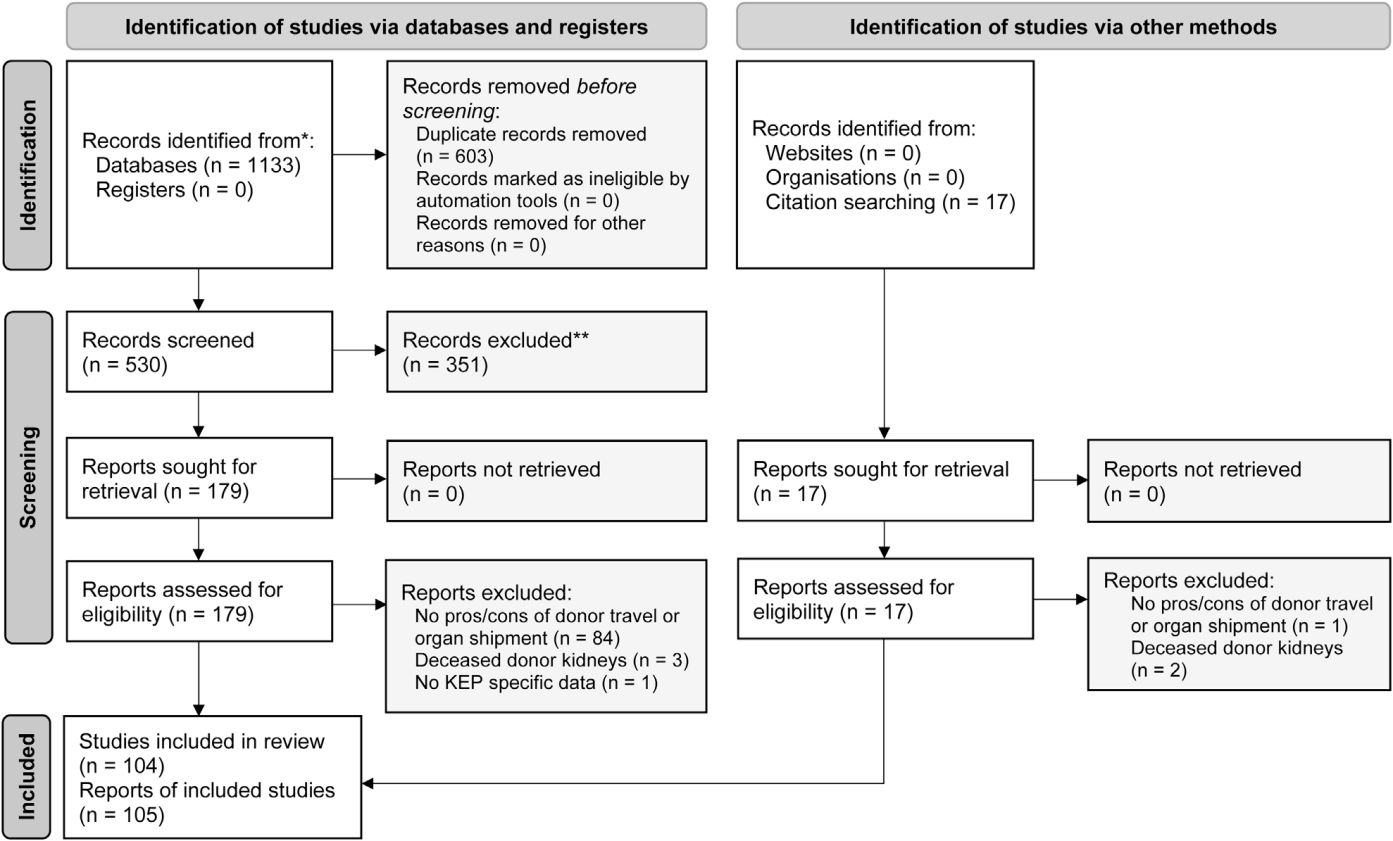
PRISMA flow diagram of the systematic search and review of donor travel and organ shipment in kidney exchange programs, *adapted from Page et al.* [28].

Our initial search identified 530 unique publications, of which 91 were included after full text screening (Figure 1; Supplementary Table S2). An additional 14 articles were found through citation checking of included studies. The majority of included studies were from the United States (63%) and Canada (13%). Additionally, we searched for the characteristics of 22 multicenter KEPs. For ten KEPs, we found the data on the Internet. Of the twelve KEPs that were contacted, nine provided us with data on their program.

### Current KEP Practices

Worldwide, multicenter KEPs vary substantially in size and contribution to the national living donor kidney transplant program (Table 1). Organ shipment is the predominant modality in 15 of 22 described programs. India, Saudi Arabia and the Netherlands reported donor travel [32, 46], while Canada reported a recent transition from donor travel to organ shipment after the COVID-19 pandemic [47]. KEPs in the United States (US) offer a dual modality based on donors’ and recipients’ preferences. [48–52].

**TABLE 1:** Characteristics and annual volume of multicenter kidney exchange programs worldwide.

Kidney exchange program	Organ shipment/donor travel	Annual KEP transplants in 2023 (% of living donation)
Australia and New Zealand Kidney Exchange	Organ shipment [29]	74 (22%) [30]
Austria and Czech Republic and Israel	Organ shipment [31]	3 (3%) [30]
Belgium	Organ shipment^a^ [32]	9 between 2013–2023^a^ [33]
Canada	Both (organ shipment in 72% in 2023) [34]	100 (±25%) [34]
France	Organ shipment^b^	4 (1%) in 2022^b^
India	Donor travel preferred in guideline [35]	198 (2%) total KEP transplants, including single center programs [30]
Italy	Organ shipment [32]	11 (3%) [30]
Netherlands	Donor travel [32]	31 (6%) [36]
Poland	Both shipment, donor travel and recipient travel^c^	1 (1%)^c^
Portugal	Organ shipment [32]	3 (4%) [30]
Saudi Arabia	Donor travel^d^	2 (national KEP started in 2024)^d^
ScandiaTransplant Exchange Program	Organ shipment [32]	17 (6%) [37]
Slovakia	Both^e^	3 (1%) between 2014–2024^e^
South Alliance for Transplants (Portugal, Italy, Spain)	Organ shipment^f^	3^f^
South Korea	*No data available upon request*	*No data available upon request*
Spain	Organ shipment^f^	16 (4%)^f^
Switzerland	Organ shipment^g^	2 (2%) [38]
Turkey and Kirghizia	Donor travel [39]	3 in 2013 [39]
United Kingdom Living Kidney Sharing Scheme	Organ shipment [40]	199 (24%) in 2023–2024 [41]
United States	Both	1282 (19%) [42]
Alliance for Paired Donation	Organ shipment [43]	*No data available upon request*
National Kidney Registry	Both (shipment in 85% from 2008–2017) [44]	19 (excluding 198 voucher and 9 unspecified donations) [45]
United Network for Organ Sharing	Mainly organ shipment^h^	15^h^

KEP, kidney exchange program.

Annual KEP volume is based on cited references or on personal communications:

^a^ Personal communication (Prof. dr. H. de Fijter and N. Mauws, 2024, e-mail).

^b^ Personal communication (P. Hesky, 2024, e-mail).

^c^ Personal communication (Dr. D. Kamińska, 2024, e-mail).

^d^ Personal communication (Dr. A. Al-Abadi, 2024, e-mail).

^e^ Personal communication (Prof. dr. I. Dedinská, 2024, e-mail).

^f^ Personal communication (Dr. B. Domínguez-Gil, 2024, e-mail).

^g^ Personal communication (Prof. dr. P. Ferrari and L. Straumann, 2024, e-mail).

^h^ Personal communication (A. Paschke, 2024, e-mail).

### Cold Ischemia Time

Organ shipment has the disadvantage of prolonging CIT [18, 53]. As many studies had overlapping cohorts [44, 48, 54–56], original studies with a head-to-head comparison of shipment versus donor travel in KEP were limited [19, 57–59]. We therefore extrapolated the analysis with circumstantial evidence (e.g., KEP versus non-KEP) and categorized studies per type of comparison.

#### Shipped Versus Non-Shipped Grafts

Nine studies compared DGF incidence in shipped versus non-shipped grafts, mostly including KEP transplants only, while Serur et al. included non-KEP controls (Supplementary Table S3) [19, 44, 48, 51, 54–59]. Four studies reported data on unique cohorts [19, 51, 57–59]. Analysis of the US transplant registry revealed a higher DGF incidence (4.5% vs. 3.3%) in 772 shipped grafts (median CIT 8 h) versus 1,651 non-shipped KEP grafts (CIT not reported), although this did not remain statistically significant in a multivariate model (OR 1.40, 95% CI 0.88–2.40) [59]. Regarding graft survival, no association was found between organ shipment and all-cause (HR 0.89, 95% CI 0.62–1.30) or death-censored graft failure (HR 0.70, 95% CI 0.46–1.08) in a Cox multivariate model [59]. Two case series reported DGF in 2/84 and 1/11 shipped grafts, versus 0/16 and 0/9 in non-shipped KEP grafts, respectively [19, 57, 58]. In contrast, Serur et al. reported comparable DGF incidence for shipped KEP versus non-shipped living donor transplants in the US. [51].

#### KEP Versus Non-KEP Transplants

Six studies compared KEP to non-KEP transplants, with on average longer CIT in the KEP group, but no reported shipping or travel status (Supplementary Table S4) [18, 43, 60–63]. A longer CIT (median 8.8 versus 1.0 h) and higher adjusted DGF incidence (adjusted OR 1.36, 95% CI 1.05–1.75) were reported for National Kidney Registry (NKR) transplants compared to control living donor transplants in the US. A cohort study in the United Kingdom (UK) found longer median CIT (339 versus 182 min) and higher DGF incidence (5.7% versus 2.9%, p < 0.001) in 1,362 KEP compared to 7,909 non-KEP transplants [18]. In adjusted logistic regression with KEP transplants only, DGF risk was higher for prolonged CIT (coefficient −0.59 for CIT <339 versus >339 min, p = 0.04). All six studies did not find significant differences in patient or graft survival nor in acute rejection rates (Supplementary Table S4).

#### Shipped Transplants Without Control Group

Fifteen studies examined shipped transplants without non-shipped controls (Supplementary Table S5) [11, 15, 64–76]. A US study analyzing 1,698 shipped grafts found a significantly higher mean CIT in grafts with DGF compared to grafts without DGF (9.0 vs. 6.8 h, p = 0.04) [69]. Another US study compared 2,364 functioning grafts and 38 early lost grafts (≤1 year) and reported no difference in CIT (8.8 vs. 8.8 h) [74].

#### Long Versus Short CIT in Living Donor Transplants

Four studies compared CIT intervals in living donor transplants in general (Supplementary Table S6) [54, 77–79]. Van de Laar et al. (2022) [78] pooled five studies [17, 59, 61, 80, 81] in a meta-analysis, comparing CIT <4 h to CIT >4 h regardless of shipping. There was a significantly lower DGF incidence for CIT <4 h (OR 0.61, 95% CI 0.49–0.77) [78]. Survival data showed a significantly lower death-censored graft survival after 1-year (OR 0.72, 95% CI 0.60–0.87) and 5-year (OR 0.88, 95% CI 0.79–0.99) for grafts with CIT >4 h in univariate analysis. Another meta-analysis showed a pooled mean difference of 21 min CIT (95% CI 6–36 min) between living donor transplants with and without DGF [79]. Notably, one of the included studies reported a significantly longer shipping distance for DGF cases as well (mean 21.8 versus 15.7 miles, p = 0.033) [69].

### Logistics

Feasibility of organ shipment depends on the local infrastructure [16, 82]. In most countries, extensive experience exists with shipping deceased donor kidneys [83]. Studies therefore recommend leveraging the existing Organ Procurement Organization (OPO) infrastructure for packaging and transport (Table 2) [15, 85, 86, 88–92].

**TABLE 2:** Expert and consensus reports about the logistics and billing of care in organ shipment and donor travel.

Study and Country	Study type	Participants	Results
Mast et al, 2011 [84] United States	Consensus report based on multiple phone conferences	N = 9 Representatives from nine medical centers	- The consensus financial model has seven principles - The model is currently used by over fifty transplant centers participating in the National Kidney Registry in the United States. Afterwards, no transplants have been cancelled anymore due to financial reasons
Irwin et al, 2012 [85] United States	Statement and proposal	N = 3 Representatives from three major commercial health payers in the United States	- Donor charges should be billed to the recipient’s center by the OPO. Donor costs and evaluation are standardized: standardized laboratory testing, standardized administration fee for the matching program, and standardized organ acquisition charges - Existing OPOs should manage organ acquisition logistics, transportation, and financial transactions in the same way they manage deceased donor organs today
Melcher et al, 2013 [86] United States	Consensus conference report	N = 73 Transplant hospital personnel, transplant recipients and donors, insurance industry and government agency representatives	- A national KEP standard acquisition charge would best achieve the criteria for a financial model - Packaging, labeling and transportation may benefit from OPO support or guidance. A logistical call should confirm the dates, operating room time and details of kidney transportation. Direct surgeon-to-surgeon communication is recommended prior to and immediately after KEP donor nephrectomy. All kidney transports should follow chain-of-custody principles. When traveling by commercial plane, all flights should be designated lifeguard. Kidneys on non-stop routes should be accompanied by a tracking device. Kidneys on routes involving any layovers should be accompanied by a courier
Ellison, 2014 [52] United States	Systematic review and case studies based on interviews	N = 4 Representatives from transplant centers and KEPs in the United States	- The main rationale for transplant centers employing their own KEP program is to avoid the logistical complexities associated with shipping kidneys - Reimbursement for surgical services is an added complexity associated with KEP. Healthcare costs can vary considerably between centers. It is often much less costly to perform matches internally - A streamlined logistical process, led by the transplant program, with strict guidelines, dictated timetables and scheduled conference calls is preferred by transplant coordinators
Tietjen et al, 2019 [87] United States	Consensus report and guidance	N = 7 Experts in transplant administration and clinical care	- For shipment, the donor hospital bills the recipient’s hospital for procurement and transportation costs. Donor and recipient’s hospital record the acquisition costs on the Medicare Cost Report, specific for the donor hospital offset by received payments from the recipient’s hospital - For donor travel, the hospitalization costs should be included on the Medicare Cost Report of the recipient’s transplant program

KEP, kidney exchange program; OPO, organ procurement organization.

Most studies report the use of commercial airlines and couriers for shipment [15, 19, 44, 48, 54–56, 58, 66, 67, 89, 91]. Mostly, kidneys are unaccompanied during flights [89, 91], but they should be accompanied by couriers during layovers to arrange alternative transportation in case of delays or missed connections [86]. Direct flights are preferred whenever available [15]. To minimize delays at the airport, some countries use “lifeguard status”, i.e., flight control provides priority for take-off, landing and unloading for commercial flights with kidneys on board [55, 86]. Private jets may be used to reduce the risk of delays [15, 44, 55, 67, 68, 72, 91], though at significantly higher costs compared to commercial flights (US$30,000 versus US$300 –US$550, respectively) [55, 65, 88]. Global Positioning System devices have been proven useful in monitoring transport progress and locating misrouted kidneys [46, 51, 55, 65, 84, 89–91, 93, 94].

Due to the complex logistics [13, 15, 44, 48, 55], hospitals rely on experienced transplant coordinators to oversee the process [19, 50, 51, 58, 72]. Some KEPs organize structured conference calls to review standardized checklists, set up guidelines for transport and coordinate the timetable [48, 57, 95]. This “transplant-program-led” approach is preferred by transplant coordinators (Table 2) [52, 86]. To ensure good cooperation, studies recommend surgeons to discuss donor anatomy and surgical aspects, packaging and cold storage solution, and surgery times in advance, and to verify recipient’s status shortly before nephrectomy [15, 19, 48, 50, 55, 58, 86].

Scheduling the surgeries is challenging: hospitals should take into account the time for donor nephrectomy, organ preparation and packaging, transport, and the expected interval between arrival and implantation [17, 19]. In addition, organ shipment can shift elective transplant procedures to out-off-office hours in case of long shipping distances or unexpected delays [12, 17, 24, 65, 86, 96, 97], especially when shipping across time zones [65]. An advantage of organ shipment is the ease of maintaining anonymity during hospitalization [12, 92, 98].

No logistical, hazardous events have been reported that directly led to transplant cancellation or graft loss, except for a single case of primary non-function possibly linked to packaging issues [99]. In the NKR, some kidneys were mistakenly left off scheduled flights, but were quickly retrieved with tracking devices and flights rescheduled [93]. Nonetheless, transport delays remain a risk in organ shipment [15, 51, 86]. Unforeseen events can extend CIT, for example, travel congestion, flight delays, weather disruptions, intra-operative delays, and after-hours emergencies affecting surgical staff or operating room availability [17, 19, 24]. In Australian KEP, re-scheduling of flights was required in 19 of 100 cases due to variation in the duration of donor nephrectomy, resulting in two delayed shipments and 17 shipments with earlier flights [19].

In recent years, several international exchanges have been performed [15, 32, 68, 70, 72, 100]. However, logistical difficulties have posed a great challenge in these international collaborations [46, 55, 72, 101, 102]. Different languages, protocols, laws, reimbursement policies, and custom clearance must be overcome [68]. Especially, international travel of donors can cause difficulties, due to the complex KEP logistics and unpredictable timeframe [102]. A study describing a transatlantic, global exchange between the Philippines and the US reported challenges with visa and immigration requirements, transmissible diseases, funding for lodging, follow-up care and donor complication insurance [103].

#### Billing

Donor evaluation and organ procurement costs need to be charged to the matched recipient’s center or insurance provider if costs cannot be charged to the intended recipient’s payor, such as for UDs, and cannot be reimbursed by the donor insurance [87]. However, variation in these costs between centers led to delayed transplants and hampered kidney exchange in general in the US. [40, 50, 52, 84, 85, 95]. Financial disincentives for centers towards KEP participation also extend to donor travel: when the UD travels to a different center for donation, the referring center incurs evaluation costs but does not receive a donor kidney in return [13].

To overcome these financial barriers, several models have been developed in the US. One approach involves transactions being channeled through OPOs, comparable to deceased donation [57, 85], by using a standardized acquisition charge. This model is preferred by transplant professionals and commercial payers in the US (Table 2) [85, 86]. Alternatively, the NKR has developed a model that relies on Medicare cost reports for billing, with the recipient center being financially responsible for the shipment [84, 104].

#### Donor Care in Different Centers

Donor travel comes with additional evaluation costs [21, 22, 24, 65, 86], as both the referring and transplanting centers assess the donor’s suitability to donate [86, 99, 105]. Variations in donor acceptance criteria between centers may result in the decline of proposed matches (Table 3) [99]. Furthermore, traveling donors receive care from two different transplant teams [12, 46, 106], which may lead to greater inconsistencies in donor counseling (Table 3). In Canadian KEP, proposed surgery at the referring hospital differed from eventual surgery in the transplanting hospital in 31%, of which 50% were significant deviations in surgical approach, such as laparoscopic to open or right to left side [21].

**TABLE 3:** Discrepancies between centers in donor evaluation when the donor travels for kidney exchange.

Study and country	Inclusion	Results
Cole et al, 2015 [99], Canada	439 KEP candidates and 467 KEP donors	240 transplants were completed, while 58 proposed matches were declined. The transplanting center declined donors that were approved by the referring center due to medical reasons in 19, due to surgical reasons in three, and due to non-medical reasons in 11 donors
Reikie et al, 2017 [21], Canada	51 KEP donors with surgical work-up and nephrectomy in different centers	Performed donor nephrectomy in the transplanting center differed from the initially proposed surgery in the referring center in 16 of 51 cases (31%). For donors with different surgery performed than proposed, three had surgery on the opposite side. Four had an open procedure instead of a laparoscopic procedure. Other conversions included open to laparoscopic (n = 3), and hand assisted to laparoscopic (n = 2) or laparoscopic to hand-assisted nephrectomy (n = 6)

KEP, kidney exchange program.

### Donor/Recipient Perspectives

Travel to the recipient’s center is often described as an inconvenience for donors [12, 13, 15, 21, 22, 24, 47, 51, 89, 93, 95–97, 107–110]: travel to a distant city, surgery in an unfamiliar hospital with unfamiliar staff, being separated from the intended recipient and social support system, incurring costs for travel and lodging, and discontinuity of care and follow-up may reduce a donor’s willingness to participate in KEP. For large geographical distances and different language regions, travel may even be a major hindrance [12, 110–112]. Donor travel may be especially inconvenient for compatible pairs, which could have donated directly to their intended recipient without the emotional distress and logistical complexity of travel [65, 113]. However, a US simulation study suggested that most compatible pairs included in a national KEP pool could be matched within their own center, minimizing the need for travel [114].

Multiple studies have stated that organ shipment contributed to the expansion of the KEP donor pool in the US [89, 93, 94, 115–117] and that shipment was preferred by KEP participants [57, 66, 90, 91]. In interviews, travel and additional travel expenses were mentioned by donor candidates as barriers for KEP participation (Table 4) [118–120]. Survey studies have found that donor travel to another region decreases willingness for compatible KEP participation (Table 5) [20, 119, 121, 122].

**TABLE 4:** Donor and recipient perspectives on donor travel and travel expenses.

Study and country	Participants	Results of interview studies
Kranenburg et al, 2006 [118] Netherlands	N = 96 24 directed and 24 KEP donor candidates and their intended recipients	- Most often, emotional reasons were mentioned as reasons not to participate in KEP. Other reasons not to participate were practical objections, for instance, if the donor had to travel to another hospital
Fortin et al, 2021 [119] Canada	N = 35 18 donor and 17 transplant candidates for compatible living kidney transplantation	- Major concerns for KEP expressed during interviews were: no emotional bond with donor/recipient, fear of broken chains or donor reneging, delays in transplantation, additional travel and related costs
		- Donors were reluctant to travel to the recipient’s center, because they want to stay close to family for support and do not want to deal with an unfamiliar medical team with which they have not yet established trust - Reimbursing travel expenses for a traveling companion to have support during organ recovery and offset lost income were cited as facilitating factors for KEP participation
Maghen et al, 2021 [120] United States	N = 31 Secondary analysis of telephone interview and questionnaire in previous non-directed donors	- 20 participants (65%) discussed financial concerns during the interviews, while 11 participants stated they were not concerned about costs (35%). Donors with financial concerns were younger (mean age 44 versus 54, p = 0.01) - Direct costs (travel, lodging, parking) were mentioned by 11 participants, with the majority about travel to and from the transplant center

KEP, kidney exchange program.

**TABLE 5:** Impact of donor travel on the willingness of donors and recipients to participate in kidney exchange.

Study and country	Participants	Survey question		Reported willingness		
				Less willing	No change	More willing
Ratner et al, 2010 [119], United States	N = 105 Survey of 53 donor and 52 transplant candidates at initial evaluation visit in the out-patient clinic	Willing to participate in altruistic unbalanced paired kidney exchange?	Donors	*Mean Likert score* ^a^ *3.1*		
			Recipients	*Mean Likert score* ^a^ *3.4*		
		Willing to participate if the donor must go to another hospital than the recipient?	Donors	*Mean Likert score* ^a^ *3.2*		
			Recipients	*Mean Likert score* ^a^ *3.3*		
Hendren et al, 2015 [20], Canada	N = 116 Survey of 81 previous living directed donors and 35 recipients who responded to be willing to participate in KEP if this option had been provided at the time of donation	The donor was required to travel out of province	Donors	51%	47%	3%
			Recipients	19%	76%	5%
		Reimbursements of travel expenses for me and traveling companion were provided (currently only donor expenses are reimbursed)	Donors	0%	28%	72%
Kute et al, 2017 [122], India	N = 300 Survey of patients with end-stage kidney disease who consented to KEP transplantation	Willing to travel to other centers in multicenter KEP	Recipients	*50% not willing due to disparity in quality and cost of healthcare*		
Fortin et al, 2021 [119], Canada	N = 116 and N = 111 Survey of 116 donor and 111 transplant candidates undergoing evaluation for compatible living kidney donation	The donor must go to another hospital for surgery but stayed in the same city	Donor	7.8%	83.6%	8.6%
			Recipient	8.1%	81.8%	10.8%
		The donor must travel to another province to donate	Donor	36.2%	58.6%	4.3%
			Recipient	28.3%	62.6%	8.1%
		Travel expenses for the donor and one travel partner are covered if they must travel to another province to donate	Donor	2.6%	31.0%	66.4%
			Recipient	0%	23.4%	76.6%
		Travel expenses for the donor and >1 travel partner are covered if they must travel from another province	Donor	2.6%	57.8%	36.7%
			Recipient	1.8%	35.1%	63.1%
		Logistics of donor travel as the most important factor that would hinder my decision to participate	Donor	*6/116 (5%)*		
			Recipient	*12/111 (11%)*		
		Upfront costs of traveling as the most important factor that would hinder my decision to participate	Donor	*4/116 (3%)*		
			Recipient	*12/111 (11%)*		

KEP, kidney exchange program.

^a^ Likert score 1=strongly disagree, 2=disagree, 3=neither agree nor disagree, 4=agree, 5=strongly agree.

In the US, some KEPs take donor travel preferences and restrictions into account when matching [52]. While this approach respects individual preferences, it can significantly impact match rates. Two simulation studies on a national US KEP showed that pairs willing to travel outside of their region had more and better quality matches and shorter waiting times [123, 124], especially for difficult-to-match pairs [123].

#### Travel Expenses

Traveling donors often pay upfront for transportation, fuel, parking, food and accommodation for themselves and a traveling companion. Although these costs may be reimbursed later, the initial expenses can be of concern. In interviews, UDs and donor-recipient pairs expressed concerns about the costs of travel (Table 4) [120]. Donors reported increased willingness to participate in KEP if travel expenses were reimbursed for both themselves and traveling companion (Table 5) [20, 119].

Currently, provincial governments reimburse travel expenses in Canada [40, 99]. However, Canadian KEP donors faced high travel expenses and a significant financial gap of 1,677 Canadian dollars despite this reimbursement (Table 6) [125, 126]. In the US, recipients are permitted to cover their donor’s travel costs [22, 115, 127]. The National Living Donor Assistance Center provides reimbursements if expenses cannot be reasonably covered by governments or insurance providers and the recipient experiences financial hardship. In Iran, reimbursements are funded through charitable donations and contributions from KEP participants within the exchange chain [128]. In Europe, Biro et al. [32] reported that countries with the most developed KEPs have cost neutral reimbursement policies.

**TABLE 6:** Travel costs for kidney exchange donors reported in prospective surveys.

Study and country	Inclusion	Included costs	Results
Przech et al, 2018 [125], Canada	676 living directed donors, 111 KEP donors and 34 UDs	Ground and air travel, parking, accommodation, prescription medications	Median out-of-pocket costs were 1,254 CAD for direct living donors, with mean difference of +205 CAD for KEP donors and −316 for UDs (both not significant)
Barnieh et al, 2019 [126], Canada	137 directed, 14 KEP donors and 8 UDs in Ontario that received reimbursements from a reimbursement program	Ground and air travel, parking, accommodation, prescription medications	Mean out-of-pocket costs were 2,212 CAD and mean amount reimbursed was 925 CAD for all living donors. KEP donors and UDs had a mean gap of, respectively, 1,677 CAD and 2,691 CAD between out-of-pocket costs and reimbursements

CAD, canadian dollars; KEP, kidney exchange program; UD, unspecified donor.

### Professional Perspectives

Many transplant professionals have expressed concerns about potential negative effects of shipping on graft outcomes [12, 55, 66, 78, 83, 89, 90, 105, 129–131], the complex logistics of multi-center KEPs [26, 52, 53, 94, 106], and the burden of travel for donors (Table 7) [49, 105, 107, 132, 133]. Good outcomes after shipment encouraged professionals to start shipping organs [13, 116, 117]. Consensus reports in the US stated that UDs should not be burdened by donor travel [105], living donor kidneys could be shipped safely [86], and that organ shipment would enhance KEP participation [86]. Recently, Canadian transplant surgeons reached consensus on shipping kidneys whenever possible, to eliminate the disincentive of donor travel [47]. Similarly, Australia mandated shipping to ensure consistent donor care and clarity of expectations about the donation process [19].

**TABLE 7:** Professional perspectives on donor travel and organ shipment in kidney exchange programs.

Study and Country	Study type	Participants	Results
Adams et al, 2002 [105] United States	Report of National Conference	N = 32 American transplant professionals (medical, logistical, government)	- Donor travel is ideal from surgical perspective due to short CIT and low DGF rate - UDs are at risk of non-reimbursed expenses due to limited available financial resources. UDs should not be burdened to travel
Woodle et al, 2005 [132], United States	Survey prior to initiation of multicenter KEP	N = 48 Transplant program personnel from eight transplant programs	- A significant degree of indecisiveness was expressed (mean Likert score 2.7) about the decision to participate in multicenter KEP. - Specific concerns and perceived barriers to multicenter KEPs included: (1) the need for donor travel, (2) financial concerns, (3) privacy and confidentiality maintenance, (4) medical equity assurance of quality of kidneys and (5) potential for medical-legal complications
Woodle et al, 2005 [49] United States	Pre- and post-conference survey	N = 48 Representatives from eight transplant programs	- Mean Likert score^a^ (1 = strongly agree, 5=strongly disagree) for being concerned about travel costs for the donor was 1.7 before and 1.49 after the educational conference (no significant difference)
Clark et al, 2010 [107] United States	Web-based survey	N = 78 Directors of 78 different transplant programs	- Donor travel was frequently cited in the open-ended comments by centers that did not want to participate in national KEP. - Logistics of donor travel was the most frequently cited, but not most important, barrier to national KEP participation
Durand et al, 2014 [133] Canada	Semi-structured interview study	N = 19 Transplant personnel from four adult transplant centers	- Traveling companion expenses for compatible pairs should be reimbursed if organ shipment is not possible - Transporting the kidney rather than the donor was one of the four conditions mentioned for compatible pair participation
Melcher et al, 2013 [86] United States	Consensus conference report	N = 73 Transplant hospital personnel, transplant recipients and donors, insurance industry and government agency representatives	- Donors should have the option, but never be required to travel to the recipient’s center. KEP centers should be willing to transport kidneys, both from and to the center, as current evidence shows it can be performed safely and it maximizes KEP participation and volume - Priorities for reducing distance between centers and prioritizing same center matches could be incorporated but should be deemphasized, as they represent logistical rather than biological considerations - Payers should cover donor travel and lodging costs when a donor travels for KEP.
Tietjen et al, 2019 [87] United States	Consensus report and guidance	N = 7 Experts in transplant administration and clinical care	- Transplant programs should facilitate reimbursement of travel costs by referring donors to the available services, including insurance providers and the National Living Donor Assistance Center

CIT, cold ischemia time; DGF, delayed graft function; KEP, kidney exchange program; UD, unspecified donor.

^a^ Likert score 1=strongly agree, 2=agree, 3=neither agree nor disagree, 4=disagree, 5=strongly disagree.

Some studies have suggested that surgical issues may arise when a kidney is procured and transplanted by different teams in organ shipment. The implanting surgeon cannot customize the donor nephrectomy to the specific needs of the recipient and relies on the donor surgeon to receive a transplantable organ [19, 50, 54]. This requires a high level of trust in the quality of the external donor nephrectomy [66, 74]. Reassuringly, in the Australian KEP, concerns from recipient surgeons about donor procurement quality were uncommon [19].

## Discussion

Multicenter KEPs face a fundamental choice: whether to ship the donor kidney or let the donor travel. The decision hinges on balancing the medical safety and logistical challenges of shipment with the burden of travel and potential disruptions to donor care. As KEPs gain prominence in optimizing living donation programs, addressing this dilemma is crucial in all (new) KEPs.

An important, medical argument against organ shipping is the prolongation of CIT. Current studies comparing shipped to non-shipped grafts, KEP to non-KEP transplants or CIT intervals within KEP do not reveal a significant impact of shipment on graft survival. However, a meta-analysis comparing short and prolonged CIT in living donor kidney transplants, irrespective of shipping, found impaired graft survival for prolonged CIT [78]. Graft survival in these type of studies may be biased by prolonged surgery duration: the prolonged CIT group had more markers of transplant complexity, such as re-transplantation and sensitization, and included in-center procedures without organ shipment [81]. These transplant complexity factors have also been associated with DGF [79]. Nonetheless, shipment itself and shipping distance have been associated with DGF in other studies, though the absolute increase was small and not significant after adjustment in Gill et al. [59, 69] Limitations in study design, lack of sufficient adjustment of confounding factors, and significant heterogeneity between studies in CIT duration and local care practices, prevent drawing robust conclusions on the safety of CIT extension. Current evidence does not support a specific cut-off for safe CIT prolongation. It is therefore recommended to keep CIT as short as possible, without compromising transplant opportunities. Comprehensive analysis of data on the safety of shipment is warranted, especially for Europe with the current collaboration for European KEP programs.^1^

Next to CIT, certain patient characteristics might influence the medical risks of shipping, such as donor age, recipient’ body mass index, or sensitization [79, 134]. This is an important consideration when shipping over long distances: it might benefit highly immunized patients by expanding the donor pool [18, 68, 98, 101], but these immunized patients are likely more susceptible to the adverse effects of prolonged CIT. Continuous hypothermic machine perfusion during transport might be useful in cases with high risk for DGF or graft loss [18, 134, 135], as it has been demonstrated to reduce DGF and improve 1-year graft survival in deceased donor kidneys [136]. KEPs could consider including the expected CIT in the allocation algorithm [16, 18, 78], although this might aggravate disparities between KEP participants [86].

To overcome the logistical challenges of shipment, KEPs could cooperate with OPOs: they have experienced coordinators, agreements with logistical partners, guidelines for transport and support for billing. We recommend scheduling conference calls between both centers with standardized checklists, as is practiced in the NKR [48], to facilitate communication about surgical and logistical issues. To avoid prolongation of CIT due to logistical barriers, centers should ensure operation room and staff availability and track logistical delays [137]. However, waiting for the arrival of a shipped kidney might be a major challenge for centers with tight operation room scheduling. Furthermore, delayed arrival of the kidney requires additional surgical staff during out-of-office hours.

Donor travel eliminates the medical risks and logistical complexity of shipping [24]. In addition, it enables the surgeon to perform both nephrectomy and implantation, which might be preferred by some centers. In countries with limited resources or limited logistical infrastructure, donor travel might be more convenient for transplant professionals and less costly. For the traveling donor, however, a high number of inconsistencies between centers in donor evaluation and counseling has been reported [21, 99]. In case of donor travel, both centers review the safety for the donor and the quality of the kidney, while in organ shipment the transplanting center mainly reviews the quality of the kidney (as in deceased donor allocation). The double donor evaluation in case of door travel increases evaluation costs, is prone for inconsistencies and likely reduces donor convenience. For example, Canadian living kidney donors reported frustration with the duplication of tests and poor information exchange between centers [139].

Disparities in healthcare quality between centers discourage donors to travel to another center [122]. However, this also hampers organ shipment, as the recipient surgeon must rely on the donor surgeon for the kidney procurement. Due to this dependency, transplant surgeons might feel reluctant to accept surgical-technical challenging or extended-criteria kidneys. It is necessary to standardize and disseminate KEP protocols, especially in international KEPs, for donor evaluation, informed consent, surgery and follow-up [139].

Donors reported reduced willingness to participate in KEPs when traveling to another region. Remarkably, willingness was not reduced if they had to travel to another hospital in the same city, suggesting that the unfamiliarity with the other hospital and team might not be a main hurdle [119]. In the Dutch KEP with donor travel, graft outcomes and health-related quality of life were similar for KEP and non-KEP donors [135, 140], although this could be related to the relatively short travel distances.

Most of the logistical and financial distress of donor travel can be addressed by good reimbursement programs and consistent donor evaluation and counseling. Healthcare payors should therefore provide reimbursements for all out-of-pocket costs of KEP donors and traveling companion, including travel, parking, accommodation, meals, and loss of workdays, also in cases where the recipient center declines the traveling donor after evaluation [50, 86, 96, 141]. In addition, centers should manage expectations of traveling KEP donors: the decision for surgery type and side of nephrectomy should be left to the operating donor surgeon. Counseling of potential donors must be improved, as only half of all donors in the NKR received education about organ transport and reimbursements [142]. Combined policies with both organ shipment and donor travel based on donor/recipient preferences can be considered to optimize donor convenience.

### Strengths and Limitations

This review summarizes current evidence on organ shipment and donor travel in KEP, providing actionable recommendations for policymakers and clinicians (Table 8). KEPs should weigh these arguments for their specific situation.

**TABLE 8:** Recommendations for clinical practice.

Organ shipment	Donor travel
Keep CIT as short as possible without compromising transplant opportunities, given the potentially higher risk of DGF.	Ensure comprehensive reimbursement of travel-related out-of-pocket costs for the donor and a travel companion, and donor’s loss of workdays, with the possibility of payments in advance.
Consider the use of machine perfusion for kidneys with expected CIT >8 h, kidneys from older donors and kidneys for highly immunized recipients.	Offer organ shipment to donors unwilling to travel (especially for unspecified or compatible KEP donors).
Collaborate with organ procurement organizations to streamline the logistics of shipment, and agree on transfer conditions and liability with logistical parties.	Discuss with the donor that evaluation will take place in two different centers and that the final surgical approach will be decided on in the transplanting center.
Organize conference calls with checklists to standardize pre- and post-operative communication between surgeons.	Communicate the KEP match to the donor after both centers reviewed and agreed on medical and immunological test results.
Schedule operation theatre upfront and keep operation theatre available when delays in transport occur.	Consider donor travel in specific situations, such as recipients with high DGF risk or surgical-technical issues, limited operating room availability, or insufficient logistical infrastructure.
Agree on the billing of donor evaluation and procurement costs with payors and insurance providers.	Ship kidneys in international exchange to ensure consistent care, follow-up and convenience for donors.
Consider including expected CIT as variable in the matching algorithm.	Consider allocation based on donor/recipient preferences or preferred travel-distances.
Share protocols for donor evaluation and surgery between the centers.	

CIT, cold ischemia time; DGF, delayed graft function; KEP, kidney exchange program.

Many of the included studies did not investigate our outcomes of interest as primary outcome. The retrospective design brings inherent bias, especially for the studies on CIT. Additionally, long term follow-up data on prolonged CIT in shipped versus non-shipped living donor kidneys was limited, and cost-comparison studies on donor travel versus organ shipment were not found. Furthermore, the external validity of our findings is limited due to a geographic disbalance: studies on CIT, logistics and professional perspectives were mainly performed in the US and studies on donor care and donor perspectives were mainly performed in Canada, while few studies were performed in Europe. Studies of KEPs in developing nations were even more sparse, and ethnic minorities were underrepresented in the qualitative studies [20, 119, 120, 125]. Additionally, while the recommendations were based on the available evidence, they may inherently reflect our interpretations, experiences, and professional opinions.

### Conclusion

Multicenter KEPs facilitate a timely and well-matched living donor transplant. However, the involvement of different transplant centers imposes challenges. Either by donor travel, organ shipment or combined policy, programs must guarantee medical and logistical safety, consistent care for donor and recipient and financial justice for all parties.

## Footnotes

1
https://www.hnbts.hu/euro-kep/project
