# Supplementary material for: Navigating a Quandary in Kidney Exchange Programs: A Review of Donor Travel versus Organ Shipment
Source: Transpl Int. 2025 Nov 12;38:14804. doi: 10.3389/ti.2025.14804 (PMC12646967; doi:10.3389/ti.2025.14804)
Supplement: Supplementary file 1 [file DataSheet1.pdf]

SUPPLEMENTARY MATERIAL

Supplementary Table S1: Searched databases and search queries.

Database (Platform)	Years of coverage	Search query	Records	After duplicates removed
Medline ALL (Ovid)	1946-present	(((Health Services Accessibility/ OR Program Evaluation/ OR Tissue and Organ Procurement/) AND Living Donors/ AND (Kidney/ OR Kidney Transplantation/)) OR (((kidney*) ADJ3 (exchang* OR pair* OR sharing* OR share* OR chain* OR allocat* OR swap*)) OR ((kidney) ADJ9 (living* OR alive OR live) ADJ9 (program*)) OR ((kidney*) ADJ6 (pair*) ADJ6 (donat* OR swap*))).ab,ti,kf.) AND (exp Travel/ OR exp Transportation/ OR Transportation of Patients/ OR (ship* OR logistic* OR travel* OR transport*).ab,ti,kf.) AND english.la. NOT (congres* OR abstract*).pt.	357	356
Embase	1971-present	('kidney paired donation'/de OR (('health program'/exp OR 'program evaluation'/exp) AND 'living donor'/exp AND (kidney/exp OR 'kidney transplantation'/exp)) OR (((kidney*) NEAR/3 (exchang* OR pair* OR sharing* OR share* OR chain* OR allocat* OR swap*)) OR ((kidney) NEAR/9 (living* OR alive OR live) NEAR/9 (program*)) OR ((kidney*) NEAR/6 (pair*) NEAR/6 (donat* OR swap*))):ab,ti,kw) AND ('organ transportation system'/de OR travel/de OR 'traffic and transport'/exp OR 'patient transport'/de OR (ship* OR logistic* OR travel* OR transport*):ab,ti,kw) AND [ENGLISH]/lim NOT ([Conference Abstract]/lim OR [Conference Review]/lim)	325	40
Web of Science Core Collection* (Web of Knowledge)	1975-present	TS=(((((kidney*) NEAR/2 (exchang* OR pair* OR sharing* OR share* OR chain* OR allocat* OR swap*)) OR ((kidney) NEAR/9 (living* OR alive OR live) NEAR/9 (program*)) OR ((kidney*) NEAR/5 (pair*) NEAR/5 (donat* OR swap*)))) AND ((ship* OR logistic* OR travel* OR transport*))) NOT DT=(Meeting Abstract OR Meeting Summary) AND LA=(English)	341	67
Cochrane Central Register of Controlled Trials (Wiley)	1992-present	((((kidney*) NEAR/3 (exchang* OR pair* OR sharing* OR share* OR chain* OR allocat* OR swap*)) OR ((kidney) NEAR/9 (living* OR alive OR live) NEAR/9 (program*)) OR ((kidney*) NEAR/6 (pair*) NEAR/6 (donat* OR swap*))):ab,ti) AND ((ship* OR logistic* OR travel* OR transport*):ab,ti) NOT ("conference abstract":kw OR Trial registry record:pt)	8	3
PsycINFO (Ovid)	1806-present	((((kidney*) ADJ3 (exchang* OR pair* OR sharing* OR share* OR chain* OR allocat* OR swap*)) OR ((kidney) ADJ9 (living* OR alive OR live) ADJ9 (program*)) OR ((kidney*) ADJ6 (pair*) ADJ6 (donat* OR swap*))).ab,ti.) AND (exp Traveling/ OR exp Transportation/ OR (ship* OR logistic* OR travel* OR transport*).ab,ti.) AND english.la. NOT (congres* OR abstract*).pt.	2	0
Additional Search Engines: Google Scholar** (100 top-ranked)		'kidney exchange program'|'kidney paired donation|exchange|swap'|'paired kidney donation|exchange| swap'|'kidney sharing|chain|swap' ship|shipping|logistics|travel|travelling|transport|transportation	100	64
Total			1133	530

*Science Citation Index Expanded (1975-present); Social Sciences Citation Index (1975-present); Arts & Humanities Citation Index (1975-present); Conference Proceedings Citation Index- Science (1990-present); Conference Proceedings Citation Index- Social Science & Humanities (1990-present); Emerging Sources Citation Index (2005-present).

*Exact search turned on in Web of Science Core Collection.

**Google Scholar was searched via "Publish or Perish" to download the results in EndNote.

Supplementary Table S2: Characteristics and domains of included studies

Study	Country	Type	Described domains: Cold ischemia time	Logistics	Donor perspectives	Professional perspectives
Adams et al, 2002[1]	United States	Expert/consensus report		x		x
Gilbert et al, 2005[2]	United States	Prospective cohort	x	x	x	
Segev et al, 2005[3]	United States	Simulation cohort			x	
Segev et al, 2005[4]	United States	Simulation cohort			x	
Woodle et al, 2005[5]	United States	Survey				x
Woodle et al, 2005[6]	United States	Editorial			x	
Woodle et al, 2005[7]	United States	Survey				x
Kranenburg et al, 2006[8]	The Netherlands	Interview			x	
Gentry et al, 2007[9]	United States	Simulation cohort			x	
Mahendran & Veitch, 2007[10]	United Kingdom	Review		x	x	
Simpkins et al, 2007[11]	United States	Retrospective cohort	x	x		
Terasaki, 2007[12]	United States	Commentary		x		
Waki & Terasaki, 2007[13]	United States	Retrospective cohort		x		x
De Klerk & Weimar, 2008[14]	The Netherlands	Editorial	x			x
Hanto et al, 2008[15]	United States	Retrospective cohort		x	x	
Johnson et al, 2008[16]	United Kingdom	Retrospective cohort	x			
Montgomery et al, 2008[17]	United States	Case report	x	x	x	
Butt et al, 2009[18]	United States	Case report	x	x	x	x
Rees et al, 2009[19]	United States	Case report	x	x		
Veale & Hill, 2009[20]	United States	Review		x	x	x
Axelrod et al, 2010[21]	United States	Review			x	
Clark et al, 2010[22]	United States	Survey			x	x
Lima et al, 2010[23]	Portugal	Retrospective cohort			x	
Minnee et al, 2010[24]	The Netherlands	Prospective cohort	x			
Ratner et al, 2010[25]	United States	Survey			x	
Veale & Hill, 2010[26]	United States	Review		x	x	
Akkina et al, 2011[27]	United States & Canada	Review		x		
Blumberg et al, 2011[28]	United States	Review		x		
Connolly et al, 2011[29]	United States	Letter to the editor		x		
Fortin & Williams, 2011[30]	Canada	Review		x	x	x
Gentry et al, 2011[31]	United States	Review			x	
Gentry & Segev, 2011[32]	United States	Review			x	x
Mast et al, 2011[33]	United States	Expert/consensus report		x		
Montgomery et al, 2011[34]	United States	Review				x

Segev et al, 2011[35]	United States	Survey	x	x	x	
Serur & Danovitch, 2011[36]	United States	Editorial	x	x	x	
Steinberg et al, 2011[37]	United States	Editorial			x	
Veale & Hill, 2011[38]	United States	Review		x	x	x
Wallis et al, 2011[39]	United States	Review		x	x	x
Chkhotua, 2012[40]	United States	Review			x	
Gentry et al, 2012[41]	United States	Review		x	x	
Irwin et al, 2012[42]	United States	Expert/consensus report		x		
Melcher et al, 2012[43]	United States	Retrospective cohort	x	x		
Segev, 2012[44]	United States	Review			x	x
Aull & Kapur, 2013[45]	United States	Review		x		x
Blumberg et al, 2013[46]	United States	Retrospective cohort	x	x		
Fortin, 2013[47]	Canada	Review			x	
Garonzik-Wang et al, 2013[48]	United States & Canada	Case report	x	x		
Kute et al, 2013[49]	India	Retrospective cohort				x
Melcher et al, 2013[50]	United States	Expert/consensus report		x		x
Ojo et al, 2013[51]	United States	Letter to the editor			x	
Bhargava et al, 2014[52]	United States	Retrospective cohort	x			
Durand et al, 2014[53]	Canada	Interview				x
Ellison, 2014[54]	United States	Systematic review		x		x
Glorie et al, 2014[55]	The Netherlands	Review		x		
Malik & Cole, 2014[56]	Canada	Review		x	x	
Treat et al, 2014[57]	United States	Retrospective cohort	x	x		x
Cole et al, 2015[58]	Canada	Retrospective cohort		x	x	
Hendren et al, 2015[59]	Canada	Survey			x	
Allen et al, 2016[60]*	Australia	Longitudinal cohort	x	x		
Krishnan et al, 2016[61]	Australia	Retrospective cohort	x			
Nath et al, 2016[62]	United Kingdom	Retrospective cohort	x			
Redfield et al, 2016[63]	United States	Retrospective cohort	x			
Böhmig et al, 2017[64]	Czech Republic & Austria	Case report	x	x		
Cowan et al, 2017[65]	United States	Retrospective cohort		x		
Gill et al, 2017[66]	United States	Retrospective cohort	x			
Kute et al, 2017[67]	India	Review		x		x
Kute et al, 2017[68]	India	Survey			x	
Rees et al, 2017[69]	United States & Philippines	Case report		x		
Reikie et al, 2017[70]	Canada	Retrospective cohort		x	x	
Sypek et al, 2017[71]	Australia	Retrospective cohort	x			
Allen et al, 2018[72]*	Australia	Longitudinal cohort	x	x		x
Flechner et al, 2018[73]	United States	Retrospective cohort	x	x		

Kute et al, 2018[74]	India	Review		x	x	
McGregor et al, 2018[75]	Canada	Review		x	x	
Przech et al, 2018[76]	Canada	Prospective cohort			x	
Treat et al, 2018[77]	United States	Retrospective cohort	x	x		x
Biró et al, 2019[78]	Europe	Survey		x	x	
Barnieh et al, 2019[79]	Canada	Survey			x	
D'Alessandro & Veale, 2019[80]	United States	Review		x	x	
Gentry & Segev, 2019[81]	United States	Review				x
Stepkowski et al, 2019[82]	United States	Retrospective cohort	x			
Tietjen et al, 2019[83]	United States	Expert/consensus report		x		
Valentín et al, 2019[84]	Italy, Portugal & Spain	Case report	x	x		
Furian et al, 2020[85]	Italy	Retrospective cohort	x			
Kher & Jha, 2020[86]	India	Review		x	x	
Leeser et al, 2020[87]	United States	Retrospective cohort	x			
McGregor et al, 2020[88]	Canada	Review			x	x
Nassiri et al, 2020[89]	United States	Retrospective cohort	x			
Shukhman et al, 2020[90]	United States	Review		x		
Syed & Augustine, 2020[91]	United States	Editorial		x	x	x
Verbesey et al, 2020[92]	United States	Retrospective cohort	x			x
Fortin et al, 2021[93]	Canada	Survey & Interview			x	
Hosseinzadeh et al, 2021[94]	Iran & United States	Review			x	
Maghen et al, 2021[95]	United States	Survey & Interview			x	
Van de Laar et al, 2021[96]	United Kingdom	Retrospective cohort	x			
Chipman et al, 2022[97]	United States	Retrospective cohort	x			
Ong & Kumar, 2022[98]	United States	Editorial				x
Van de Laar et al, 2022[99]	United Kingdom / The Netherlands	Systematic review	x			x
Kute et al, 2023[100]	India & United States	Review		x		
Weinreich et al, 2023[101]	Scandinavia	Retrospective cohort	x			
Böhmig et al, 2024[102]	Austria & Czech Republic	Review		x		
Francisco et al, 2024[103]	Portugal	Case report	x			
Tirtayasa et al, 2024[104]	Indonesia	Systematic review	x			
Verbesey et al, 2024[105]	United States	Retrospective cohort	x			

*Two reports of the same study.

Supplementary Table S3: The impact of cold ischemia time in shipped versus non-shipped living donor kidney transplants.

Study	Country	Study period	Registry	Inclusion	Cold ischemia time	Delayed graft function	Rejection and graft/patient survival	Kidney function
Gilbert et al, 2005[2]	United States	January 2000 – March 2004	WRTC	11 shipped and 9 non-shipped KEP grafts	Mean 243 vs 205 minutes	1 case reported in a graft with prolonged CIT.	-	3- and 6-month creatinine 1.6 and 1.6 vs 1.44 and 1.68 mg/dL.
Serur et al, 2011[36]	United States	-	NKR & National Institutes of Health	Shipped NKR and simultaneously transplanted living donor grafts	-	3.5% in NKR vs 3.6% national living donor DGF rate (no significance reported).	-	-
Allen et al, 2016 [60] & Allen et al, 2018[72]	Australia	October 2010 – May 2014	AKX & ANZDATA	84 shipped and 16 non-shipped KEP grafts	Mean 6.8 vs 2.6 hours	2 (2.4%) vs 0 cases (no significance reported).	1-year graft survival 98% vs 94%, mean difference 3.9% (95% CI -5.4 to 13.2).<br>1-year patient survival 98% vs 100%, mean difference −2.4% (95% CI −10.0 to 5.2).	Mean serum creatinine at 1 month 112 vs 105 µmol/l, mean difference 7.3 (95% CI -20.2 to 34.8)
Gill et al, 2017[66]	United States	January 2005 – October 2015	SRTR	772 shipped and 1,651 non-shipped KEP grafts, CIT >16 hours excluded	Median 8 hours for shipped kidneys	4.5% vs 3.3%, adjusted OR 1.40 (95% CI 0.88-2.40) for shipped vs non-shipped.	All-cause graft loss HR 0.89 (95% CI 0.62-1.30) and death-censored graft loss HR 0.70 (95% CI 0.46-1.08) for shipped vs non-shipped in Cox multivariate model.	-
Melcher et al, 2012[43]*		February 2008 – June 2011	NKR	47 shipped (14 coast-to-coast) and 53 non- shipped KEP grafts	Mean 12 hours for coast-to-coast shipped kidneys	0 cases reported.	-	Median 1-week creatinine 1.6 for coast-to-coast vs 1.5 mg/dL for non-shipped grafts (no significance reported).
Blumberg et al, 2013[46]*		July 2008 – July 2011	NKR and UNOS KEP pilot	13 shipped and 9 non-shipped KEP grafts	Median 12.5 vs 1 hour**	1 case in shipped graft (15.1 hours CIT)	-	-
Treat et al, 2014[57]*		July 2008 – May 2013	NKR & Los Angeles Ronal Reagan Medical Center	57 shipped NKR and 57 matched, in-center, non-shipped KEP or non-KEP grafts	Mean 12.1 hours for shipped grafts, CIT was set to 1.0 hour in non-shipped grafts	1 (1.8%) vs 0 (0%) cases, p=1.0.	No significant difference in 1-year (98% vs 98%, p=1.0) and 4-year graft survival (86% vs 71%, p=1.0) or graft loss (7% vs 7%, p=1.0).<br>No significant difference in graft survival in Cox multivariate model (HR 1.33, p=0.7).	No significant difference in mean serum creatinine till 1 year, except at 4 weeks postoperatively (1.16 vs 1.32 mg/dL, p=0.04).

Treat et al, 2018[77]*	February 2008 – November 2015	NKR & SRTR	1,267 KEP shipped, 205 KEP non-shipped and 4,800 non-KEP grafts	Median 9.3 vs 1.0 vs 0.93 hours	64 (5.1%) vs 7 (3.4%) vs 137 (2.9%) cases (p=0.001).	All-cause graft loss (7.0% vs 4.4% vs 6.4%, p<0.01) and patient survival (97.2% vs 98.6% vs 96.8%, p=0.001) were significantly different between the three groups, but death-censored graft survival was not (97.9% vs 99.0% vs 98.7%, p=0.50).	-
Flechner et al, 2018[73]*	February 2008 – February 2017	NKR & SRTR	2,037 NKR shipped and in-center exchanges	Median 8.7 hours for NKR grafts	101 (4.9%) in NKR, higher than in-center exchanges (incidence not reported).	-	-

AKX = Australian Kidney paired eXchange program; ANZDATA = Australia and New Zealand Dialysis and Transplant Registry; CI = Confidence Interval; CIT = Cold ischemia Time; DGF = Delayed Graft Function; HR = Hazard Ratio; KEP = Kidney Exchange Program; NKR = National Kidney Registry; OR = Odds Ratio; SRTR = Scientific Registry of Transplant Recipients; UNOS = United Network of Organ Sharing; WRTC = Washington Regional Transplant Consortium.

*Overlapping data with Gill et al. (2017).

**Calculated.

Supplementary Table S4: The impact of cold ischemia time in KEP versus non-KEP living donor kidney transplants.

Study	Country	Study period	Registry / program	Inclusion	Cold ischemia time	Delayed graft function	Rejection and graft/patient survival	Kidney function
Krishnan et al, 2016[61]	Australia	2007 – 2012	AKX & ANZDATA	33 KEP and 1,541 non-KEP transplants, CIT >8 hours excluded	Mean 5.12 vs 3.01 hours	0 (0%) vs 53 cases (3.4%), p=0.28.	No significant difference in all-cause graft loss (3.0% vs 7.3%, p=0.35), death-censored graft loss (0.0% vs 5.0%, p=0.19), and all-cause mortality (3.0% vs 2.9%, p=0.97).	-
Leeser et al, 2020[87]	United States	February 2008 – December 2017	NKR & SRTR	2,363 NKR and 54,497 control transplants (of which 4,635 non-NKR KEP)	Median 9 vs 1 hours (vs 1.5 hours for non-NKR KEP)	5% for NKR vs 3% for controls (adjusted OR 1.36, p=0.02).<br>3.8% for non-NKR KEP.	No difference in death-censored graft loss (log-rank p=0.2) and mortality (log-rank p=0.4) during the 7-year study period.<br>Lower graft loss in NKR compared to non-NKR KEP (p=0.03).	-
Bhargava et al, 2014[52]*		January 2009 – February 2012	Allegheny General Hospital, Pennsylvania	15 KEP and 30 traditional in-center living donor transplants	Mean 13.1 vs 3.8 hours	0 cases reported.	No difference in graft or patient survival (100% in both groups) and acute rejection rate (20% vs 10%**, p=0.35) after 1 year follow-up.	No significant difference in incidence of proteinuria (53% vs 73%**, p=0.18).
Stepkowski et al, 2019[82]*		January 2010 – January 2016	APD & OPTN/UNOS registry	223 APD KEP, 186 APD-other KEP, 110 APD LD, 2,946 OPTN all KEP and 31,512 OPTN living directed transplants	Mean 6.9 vs 6.6 vs 1.9 vs 4.8 vs 1.9 hours	3.1% vs 3.8% vs 5.5% vs 4.4% vs 3% (no significance reported).	95.1% vs 92.5% vs 91.8% vs 91.4% vs 91.9% survival with functioning graft, no significant differences between the groups (p≥0.05).	-
Verbesey et al, 2024[105]*		February 2008 – December 2021	NKR & SRTR	258 KEP and 3447 non-KEP pediatric (<18 years) living donor transplants	Median 2.4 vs 1.2 hours	4.3% vs 3.1%, not significant.	Death-censored graft failure was significantly higher for KEP in univariate (log-rank p=0.02), but not in multivariate analysis (HR 1.23, p=0.3).<br>Mortality was not significantly different in both univariate (log-rank p=0.3) and multivariate analysis (HR 1.67, p=0.2).	-

Van de Laar et al, 2021[96]	United Kingdom	January 2007 – December 2018	UKLKSS	1,362 KEP and 7,909 non-KEP adult living donor transplants	Median 339 vs 182 minutes	5.73% vs 2.91% for KEP vs non-KEP (p<0.001).<br>3.47% vs 1.95% for CIT >339 and <339 minutes within KEP group (p=0.03).<br>Adjusted regression coefficient -0.59 (p=0.04) for short CIT with prolonged CIT as reference.	No significant difference in 5-year patient survival (HR 1.20, p=0.1) for KEP vs non-KEP in case-control analysis with matched pairs.<br>No difference in 1-year (95% vs 97%, p=0.11) and 5-year graft survival (91% vs 92%, p=0.67) or rejection (13.5% vs 11.1%, p=0.43) between CIT >339 and <339 minutes within KEP group.<br>No association between 5-year death-censored graft loss and CIT in KEP in Cox proportional hazard regression model (HR 1.31 for CIT>10 hours compared to <4 hours, p=0.67).	1-year eGFR 55 vs 58 (p=0.04) and 5-year eGFR 53 vs 56 mL/min (p=0.01) for KEP versus non-KEP.<br>5-year eGFR 52.96 vs 51.75 mL/min (p=0.25) between KEP and non-KEP in additional case-control analysis with matching two non-KEP transplants to one KEP transplant.<br>5-year eGFR 50 vs 55 mL/min (p=0.02) for CIT >339 and <339 minutes within KEP group.

AKX = Australian Kidney paired eXchange program; ANZDATA = Australia and New Zealand Dialysis and Transplant Registry; APD = Alliance for Paired Donation; CIT = Cold Ischemia Time; HR = Hazard Ratio; KEP = Kidney Exchange Program; NKR = National Kidney Registry; OR = Odds Ratio; OPTN = Organ Procurement and Transplantation Network; SRTR = Scientific Registry of Transplant Recipients; UKLKSS = United Kingdom Living Kidney Sharing Scheme; UNOS = United Network for Organ Sharing.

*Overlapping data with Leeser et al. (2020).

**Calculated.

Supplementary Table S5: The impact of cold ischemia time in shipped living donor transplants (without a comparison between shipped and non-shipped or KEP and non-KEP).

Study	Country	Study period	Registry / program	Inclusion	Cold ischemia time	Delayed graft function	Rejection and graft/patient survival	Kidney function
Johnson et al, 2008[16]	United Kingdom	April 2007 – July 2008	UKLKSS	8 shipped paired donor transplants	Median 5 hours and 44 minutes	-	6/8 had follow-up, one graft failed after 3 months.	-
Montgomery et al, 2008[17]	United States	April 2007	John Hopkins Hospital & California Pacific Medical Center	3-way chain, 1 kidney shipment	8 hours	Immediate graft function.	No rejection after 1 year.	1-year creatinine 1.1. mg/dl, normal renal function.
Butt et al, 2009[18]	United States	-	NKR & Los Angeles Medical Center	1 intrastate and 3 transcontinental shipments	8, 14, 12, and 11 hours	0 cases reported.	No graft failures.	-
Rees et al, 2009[19]	United States	July 2007	APD & John Hopkins Hospital	3 kidney shipments	>8 hours in 2 of 3 shipments	-	Kidneys function well.	-
Segev et al, 2011[35]	United States	April 2007 – April 2010	30 KEP transplant centers	56 shipped KEP transplants	Median 7.2, mean 7.6 hours	0 cases reported.	-	No association between initial urine output or trend in creatinine post transplantation and CIT (p>0.05).
Garonzik-Wang et al, 2013[48]	United States & Canada	September 2009 – July 2010	McGill University & John Hopkins University	4 shipments, 2 international flights	<6 hours for both international shipments	Immediate graft function.	No graft failures.	-
Redfield et al, 2016[63]	United States	2000 - 2014	OPTN/UNOS registry	1,698 shipped transplants with and without DGF	Mean CIT 9.0 for DGF vs 6.8 hours for non-DGF (p=0.04)	Mean shipping distance 725 for DGF vs 579 miles for non-DGF (p=0.09).	-	-
Sypek et al, 2017[71]	Australia	From 2010 onwards	AKX	1 shipped KEP transplant	Transported over 3300 kilometers, no CIT reported	No delayed graft function	No graft or patient loss or rejection.	-
Böhmig et al, 2017[64]	Czech Republic & Austria	September 2016	Medical University Vienna & Institute for Clinical and	Cross-border two-way kidney exchange with kidney shipment	313 and 335 minutes	Immediate graft function.	No graft failures.	Slow recovery of graft function with vascular changes in biopsy in one recipient, but

			Experimental Medicine Prague					kidney function normalized within 2 weeks.
Valentín et al, 2019[84]	Italy, Portugal & Spain	July 2018	SAT	Two-way exchange with shipment	CIT was <8 hours	No DGF occurred.	-	Good renal function 1-week postoperatively.
Furian et al, 2020[85]	Italy	2019 - 2020	DEC-K	16 shipped living donor KEP transplants	Mean <6 hours	0 cases reported.	-	-
Verbesey et al, 2020[92]	United States	February 2009 – December 2017	NKR	2,364 NKR transplants with 38 early graft losses within 1-year post-transplant, of which 13 within 30 days posttransplant	-	-	No difference in cold ischemia time (8.8 vs 8.8 hours) between functioning and early lost grafts (≤1-year). No association between CIT>8 hours and early lost grafts (≤30 days) in multivariate linear regression (risk difference 0.003, p=0.2).	-
Chipman et al, 2021[97]	United States	October 2013 – February 2019	NKR	154 compatible recipients and 2,115 incompatible recipients transplanted via KEP	Median 10 hours for compatible recipients transplanted via KEP	1% vs 6% (p<0.001) for originally compatible versus incompatible recipients.	No differences in death-censored graft failure (log-rank p=0.7) or mortality (log-rank p=0.1) over median 3.6 years follow-up.	-
Weinreich et al, 2023[101]	Sweden, Denmark, Finland, Norway & Iceland	2019-2022	STEP	49 KEP living donor transplants, of which 8 in-center exchanges and 41 shipments, and 893 living directed transplants	Median 329 minutes	2 cases reported (4%), with CIT of 2.8 and 6.3 hours. No correlation was observed between CIT and DGF	No significant difference in two-year graft survival (95.8% versus 98.2%, p=0.35) between KEP versus non-KEP. 8 of 49 KEP recipients experienced biopsy-proven rejection <1 year post transplantation.	Median creatinine 1-year postoperatively in KEP was 98 µmol/L.

Francisco et al, 2024[103]	Portugal	March 2020 – December 2021	SAT	3 international KEP transplants in Portuguese recipients	5, 5,1 and 8.9 hours	Hypoperfusion and DGF in kidney with 8.9 hours CIT.	T-cell mediated rejection after 15 days in kidney with 5.1 hours CIT.	12-month serum creatinine 1.2, 1.1, and 1.3 mg/dL, respectively.

AKX = Australian Kidney eXchange; APD = Alliance for Paired Donation; CIT = Cold Ischemia Time; DEC-K = DECeased donor Kidney Paired Exchange; DGF = Delayed Graft Function; KEP = Kidney Exchange Program; NKR = National Kidney Registry; OPTN = Organ Procurement & Transplantation Network; SAT = South Alliance for Transplants; STEP = ScandiaTransplant Exchange Program; UKLKSS = United Kingdom Living Kidney Sharing Scheme; UNOS = United Network for Organ Sharing.

Supplementary Table S6: The impact of short versus prolonged cold ischemia time in living donor transplants.

Study	Country	Study period	Registry / program	Inclusion	Cold ischemia time	Delayed graft function	Rejection and graft/patient survival	Kidney function
Minnee et al, 2010[24]	The Netherlands	January 2000 – July 2007	Amsterdam Academic Medical Center	200 transplants, of which 12 (6%) with DGF, after hand-assisted laparoscopic living donor nephrectomy	50 transplants with CIT >180 minutes and 150 with CIT <180 minutes	6 (12%) patients with CIT >180 minutes had DGF. DGF was not associated with CIT >180 minutes in univariate (OR 3.14, p=0.08) and multivariate regression (OR 1.08, p=0.96).	-	-
Treat et al, 2018[77]	United States	February 2008 – November 2015	NKR & SRTR	6,272 living donor transplants in centers participating in the NKR	Median 9.3 vs 1.0 vs 0.93 hours	Adjusted OR 1.05 for each hour of CIT (p<0.01).	In multivariate Cox regression, CIT was not associated with 1-year death-censored (HR 1.02, p=0.40) or all-cause graft loss (HR 1.01, p=0.4) or mortality (HR 1.00, p=0.9)	-
Van de Laar et al, 2022[99]	Meta-analysis	1990-2018	Meta-analysis	Studies comparing DGF, graft and patient survival and acute rejection rate between living donor kidney transplants with CIT <4 and >4 hours	94,693 (92.65%) with CIT <4 and 7,507 (7.35%) with CIT >4 hours	DGF was 4.5% for short vs 6.8% for prolonged CIT (OR 0.61, p<0001). DGF incidence increased with CIT interval.	1-year graft survival (OR 0.72, p<0.001) and 5-year graft survival (OR 0.88, p=0.04) was lower for CIT >4 compared to <4 hours. HR for graft survival was 1.20 (p=0.006) when comparing CIT 0-2 to CIT 4-8 hours. 10-year graft survival, patient survival and acute rejection rate did not significantly differ between CIT <4 and >4 hours (p≥0.05).	-
Tirtayasa et al, 2024[104]	Meta-analysis	2000-2018	Meta-analysis	107,421 living donor transplants, of which 3,466 (3.2%) with DGF*	-	DGF was associated with longer CIT (mean difference 21.36 minutes, p=0.004).	-	-

CI = Confidence Interval; CIT = Cold Ischemia Time; DGF = Delayed Graft Function; HR = Hazard Ratio; NKR = National Kidney Registry; OR = Odds Ratio; SRTR = Scientific Registry of Transplant Recipients.

*Calculated.

REFERENCES:

1. Adams PL, Cohen DJ, Danovitch GM, et al. The nondirected live-kidney donor: ethical considerations and practice guidelines: A National Conference Report. *Transplantation*. 2002; **74**: 582-9.
2. Gilbert JC, Brigham L, Batty DS, Jr., Veatch RM. The nondirected living donor program: a model for cooperative donation, recovery and allocation of living donor kidneys. *Am J Transplant*. 2005; **5**: 167-74.
3. Segev DL, Gentry SE, Melancon JK, Montgomery RA. Characterization of waiting times in a simulation of kidney paired donation. *Am J Transplant*. 2005; **5**: 2448-55.
4. Segev DL, Gentry SE, Warren DS, Reeb B, Montgomery RA. Kidney paired donation and optimizing the use of live donor organs. *JAMA*. 2005; **293**: 1883-90.
5. Woodle ES, Boardman R, Bohnengel A, Downing K. Influence of educational programs on perceived barriers toward living donor kidney exchange programs. *Transplant Proc*. 2005; **37**: 602-4.
6. Woodle ES. The potential of paired donation programs: modeling and reality. *Am J Transplant*. 2005; **5**: 1787-8.
7. Woodle ES, Bohnengel A, Boardman R. Kidney exchange programs: attitudes of transplant team members toward living donation and kidney exchanges. *Transplant Proc*. 2005.
8. Kranenburg LW, Zuidema W, Weimar W, et al. One donor, two transplants: willingness to participate in altruistically unbalanced exchange donation. *Transpl Int*. 2006; **19**: 995-9.
9. Gentry SE, Segev DL, Simmerling M, Montgomery RA. Expanding kidney paired donation through participation by compatible pairs. *Am J Transplant*. 2007; **7**: 2361-70.
10. Mahendran AO, Veitch PS. Paired exchange programmes can expand the live kidney donor pool. *Br J Surg*. 2007; **94**: 657-64.
11. Simpkins CE, Montgomery RA, Hawxby AM, et al. Cold ischemia time and allograft outcomes in live donor renal transplantation: is live donor organ transport feasible? *Am J Transplant*. 2007; **7**: 99-107.
12. Terasaki PI. Are the effects of prolonged cold ischemia a barrier to long-distance transportation of living donor kidneys? *Nat Clin Pract Nephrol*. 2007; **3**: 368-9.
13. Waki K, Terasaki PI. Paired kidney donation by shipment of living donor kidneys. *Clin Transplant*. 2007; **21**: 186-91.
14. Klerk Md, Weimar W. Ingredients for a successful living donor kidney exchange program. *Transplantation*. 2008; **86**: 511-2.
15. Hanto RL, Reitsma W, Delmonico FL. The development of a successful multiregional kidney paired donation program. *Transplantation*. 2008; **86**: 1744-8.
16. Johnson RJ, Allen JE, Fuggle SV, Bradley JA, Rudge C, Kidney Advisory Group UKTN. Early experience of paired living kidney donation in the United kingdom. *Transplantation*. 2008; **86**: 1672-7.
17. Montgomery RA, Katznelson S, Bry WI, et al. Successful three-way kidney paired donation with cross-country live donor allograft transport. *Am J Transplant*. 2008; **8**: 2163-8.
18. Butt FK, Gritsch HA, Schulam P, et al. Asynchronous, out-of-sequence, transcontinental chain kidney transplantation: a novel concept. *Am J Transplant*. 2009; **9**: 2180-5.
19. Rees MA, Kopke JE, Pelletier RP, et al. A nonsimultaneous, extended, altruistic-donor chain. *N Engl J Med*. 2009; **360**: 1096-101.
20. Veale J, Hil G. The National Kidney Registry: transplant chains--beyond paired kidney donation. *Clin Transpl*. 2009: 253-64.
21. Axelrod DA, McCullough KP, Brewer ED, Becker BN, Segev DL, Rao PS. Kidney and Pancreas Transplantation in the United States, 1999-2008: The Changing Face of Living Donation. *American Journal of Transplantation*. 2010; **10**: 987-1002.
22. Clark E, Hanto R, Rodrigue JR. Barriers to implementing protocols for kidney paired donation and desensitization: survey of US transplant programs. *Prog Transplant*. 2010; **20**: 357-65.
23. Lima B, Dias L, Henriques AC. The Portuguese match algorithm in the kidney paired donation program. *Cells Tissues Organs*. 2010; **13**.
24. Minnee RC, Bemelman WA, Donselaar-van der Pant KA, et al. Risk factors for delayed graft function after hand-assisted laparoscopic donor nephrectomy. *Transplant Proc*. 2010; **42**: 2422-6.
25. Ratner LE, Rana A, Ratner ER, et al. The altruistic unbalanced paired kidney exchange: proof of concept and survey of potential donor and recipient attitudes. *Transplantation*. 2010; **89**: 15-22.
26. Veale J, Hil G. National Kidney Registry: 213 transplants in three years. *Clin Transpl*. 2010: 333-34.

27. Akkina SK, Muster H, Steffens E, Kim SJ, Kasiske BL, Israni AK. Donor exchange programs in kidney transplantation: rationale and operational details from the north central donor exchange cooperative. *Am J Kidney Dis*. 2011; **57**: 152-8.
28. Blumberg JM, Gritsch H, Veale JL. Kidney paired donation: advancements and future directions. *Curr Opin Organ Transplant*. 2011; **16**: 380-4.
29. Connolly JS, Terasaki PI, Veale JL. Kidney paired donation - The next step. *New Engl J Med*. 2011; **365**: 868-9.
30. Fortin MC, Williams-Jones B. Who should travel in kidney exchange programs: the donor, or the organ? *Open Med*. 2011; **5**: e23-5.
31. Gentry SE, Montgomery RA, Segev DL. Kidney paired donation: fundamentals, limitations, and expansions. *Am J Kidney Dis*. 2011; **57**: 144-51.
32. Gentry S, Segev DL. Living donor kidney exchange. *Clin Transpl*. 2011: 279-86.
33. Mast DA, Vaughan W, Busque S, et al. Managing finances of shipping living donor kidneys for donor exchanges. *Am J Transplant*. 2011; **11**: 1810-4.
34. Montgomery RA. Living donor exchange programs: theory and practice. *British medical bulletin*. 2011.
35. Segev DL, Veale JL, Berger JC, et al. Transporting live donor kidneys for kidney paired donation: initial national results. *Am J Transplant*. 2011; **11**: 356-60.
36. Serur D, Danovitch GM. Kidney paired donation: Something special in the air. *Nephrology Self-Assessment Program*. 2011; **10**: 525-9.
37. Steinberg D. Compatible-Incompatible Live Donor Kidney Exchanges. *Transplantation*. 2011; **91**: 257-60.
38. Veale J, Hil G. The National Kidney Registry: 175 transplants in one year. *Clin Transpl*. 2011: 255-78.
39. Wallis CB, Samy KP, Roth AE, Rees MA. Kidney paired donation. *Nephrol Dial Transplant*. 2011; **26**: 2091-99.
40. Chkhotua A. Paired kidney donation: outcomes, limitations, and future perspectives. *Transplant Proc*. 2012; **44**: 1790-2.
41. Gentry SE, Montgomery RA, Segev DL. Controversies in kidney paired donation. *Adv Chronic Kidney Dis*. 2012; **19**: 257-61.
42. Irwin FD, Bonagura AF, Crawford SW, Foote M. Kidney paired donation: a payer perspective. *Am J Transplant*. 2012; **12**: 1388-91.
43. Melcher ML, Leeser DB, Gritsch HA, et al. Chain transplantation: initial experience of a large multicenter program. *Am J Transplant*. 2012; **12**: 2429-36.
44. Segev DL. Innovative strategies in living donor kidney transplantation. *Nat Rev Nephrol*. 2012; **8**: 332-8.
45. Aull MJ, Kapur S. Kidney paired donation and its potential impact on transplantation. *Surg Clin North Am*. 2013; **93**: 1407-21.
46. Blumberg JM, Gritsch HA, Reed EF, et al. Kidney paired donation in the presence of donor-specific antibodies. *Kidney Int*. 2013; **84**: 1009-16.
47. Fortin MC. Is it ethical to invite compatible pairs to participate in exchange programmes? *J Med Ethics*. 2013; **39**: 743-7.
48. Garonzik-Wang JM, Sullivan B, Hiller JM, et al. International kidney paired donation. *Transplantation*. 2013; **96**: e55-6.
49. Kute VB, Gumber MR, Vanikar AV, et al. Comparison of kidney paired donation transplantations with living related donor kidney transplantation: implications for national kidney paired donation program. *Ren Fail*. 2013; **35**: 504-8.
50. Melcher ML, Blosser CD, Baxter-Lowe LA, et al. Dynamic challenges inhibiting optimal adoption of kidney paired donation: Findings of a consensus conference. *Am J Transplant*. 2013; **13**: 851-60.
51. Ojo AO, Merion RM, Howard DH, Warren PH. Response to "dynamic challenges inhibiting optimal adoption of kidney paired donation: Findings of a consensus conference" by Melcher et al. *Am J Transplant*. 2013; **13**: 2228.
52. Bhargava A, Arora S, Marcus RJ, Sureshkumar KK. Outcomes of paired-exchange live-donor kidney transplantation: a single-center experience. *Transplant Proc*. 2014; **46**: 3420-2.
53. Durand C, Duplantie A, Fortin MC. Transplant professionals' proposals for the implementation of an altruistic unbalanced paired kidney exchange program. *Transplantation*. 2014; **98**: 754-9.
54. Ellison B. A systematic review of kidney paired donation: Applying lessons from historic and contemporary case studies to improve the US model. *Wharton Research Scholars Journal*. 2014; **107**.
55. Glorie K, Haase-Kromwijk B, Klundert Jvd, Wagelmans A, Weimar W. Allocation and matching in kidney exchange programs. *Transpl Int*. 2014; **27**: 333-43.
56. Malik S, Cole E. Foundations and principles of the Canadian living donor paired exchange program. *Can J Kidney Health Dis*. 2014; **1**.
57. Treat EG, Miller ET, Kwan L, et al. Outcomes of shipped live donor kidney transplants compared with traditional living donor kidney transplants. *Transpl Int*. 2014; **27**: 1175-82.
58. Cole EH, Nickerson P, Campbell P, et al. The Canadian kidney paired donation program: a national program to increase living donor transplantation. *Transplantation*. 2015; **99**: 985-90.

59. Hendren E, Gill J, Landsberg D, Dong J, Rose C, Gill JS. Willingness of Directed Living Donors and Their Recipients to Participate in Kidney Paired Donation Programs. *Transplantation*. 2015; **99**: 1894-9.
60. Allen R, Pleass H, Clayton PA, Woodroffe C, Ferrari P. Outcomes of kidney paired donation transplants in relation to shipping and cold ischaemia time. *Transpl Int*. 2016; **29**: 425-31.
61. Krishnan AR, Wong G, Chapman JR, et al. Prolonged Ischemic Time, Delayed Graft Function, and Graft and Patient Outcomes in Live Donor Kidney Transplant Recipients. *Am J Transplant*. 2016; **16**: 2714-23.
62. Nath J, Hodson J, Canbilen SW, et al. Effect of cold ischaemia time on outcome after living donor renal transplantation. *Br J Surg*. 2016; **103**: 1230-6.
63. Redfield RR, Scalea JR, Zens TJ, et al. Predictors and outcomes of delayed graft function after living-donor kidney transplantation. *Transpl Int*. 2016; **29**: 81-7.
64. Böhmig GA, Fronek J, Slavcev A, Fischer GF, Berlakovich G, Viklicky O. Czech-Austrian kidney paired donation: first European cross-border living donor kidney exchange. *Transpl Int*. 2017; **30**: 638-39.
65. Cowan N, Gritsch HA, Nassiri N, Sinacore J, Veale J. Broken chains and reneging: a review of 1748 kidney paired donation transplants. *Am J Transplant*. 2017; **17**: 2451-57.
66. Gill J, Rose C, Joffres Y, Kadatz M, Gill J. Cold ischemia time up to 16 hours has little impact on living donor kidney transplant outcomes in the era of kidney paired donation. *Kidney Int*. 2017; **92**: 490-6.
67. Kute VB, Patel HV, Shah PR, et al. Past, present and future of kidney paired donation transplantation in India. *World J Transplant*. 2017; **7**: 134-43.
68. Kute VB, Patel HV, Shah PR, et al. Impact of single centre kidney paired donation transplantation to increase donor pool in India: a cohort study. *Transpl Int*. 2017; **30**: 679-88.
69. Rees MA, Dunn TB, Kuhr CS, et al. Kidney Exchange to Overcome Financial Barriers to Kidney Transplantation. *Am J Transplant*. 2017; **17**: 782-90.
70. Reikie BA, Kroczak T, McGregor TB. Challenges for the Travelling Donor: Variability Between Donor Workup and Donor Surgery in the Canadian Kidney Paired Exchange Program. *Transplant Proc*. 2017; **49**: 1232-6.
71. Sypek MP, Alexander SI, Cantwell L, et al. Optimizing Outcomes in Pediatric Renal Transplantation Through the Australian Paired Kidney Exchange Program. *Am J Transplant*. 2017; **17**: 534-41.
72. Allen RDM, Pleass HCC, Woodroffe C, Clayton PA, Ferrari P. Challenges of kidney paired donation transplants involving multiple donor and recipient surgeons across Australia. *ANZ J Surg*. 2018; **88**: 167-71.
73. Flechner SM, Thomas AG, Ronin M, et al. The first 9 years of kidney paired donation through the National Kidney Registry: characteristics of donors and recipients compared with National Live Donor Transplant Registries. *Am J Transplant*. 2018; **18**: 2730-38.
74. Kute VB, Prasad N, Shah PR, Modi PR. Kidney exchange transplantation current status, an update and future perspectives. *World J Transplant*. 2018; **8**: 52-60.
75. McGregor T, Sener A, Paraskevas S, Reikie B. Kidney paired donation and the unique challenges of kidney shipment in Canada. *Can J Surg*. 2018; **61**: 139-40.
76. Przech S, Garg AX, Arnold JB, et al. Financial Costs Incurred by Living Kidney Donors: A Prospective Cohort Study. *J Am Soc Nephrol*. 2018; **29**: 2847-57.
77. Treat E, Chow EKH, Peipert JD, et al. Shipping living donor kidneys and transplant recipient outcomes. *Am J Transplant*. 2018; **18**: 632-41.
78. Biro P, Haase-Kromwijk B, Andersson T, et al. Building Kidney Exchange Programmes in Europe-An Overview of Exchange Practice and Activities. *Transplantation*. 2019; **103**: 1514-22.
79. Barnieh L, Klarenbach S, Arnold J, et al. Nonreimbursed Costs Incurred by Living Kidney Donors: A Case Study From Ontario, Canada. *Transplantation*. 2019; **103**: e164-e71.
80. D'Alessandro T, Veale JL. Innovations in kidney paired donation transplantation. *Curr Opin Organ Transplant*. 2019; **24**: 429-33.
81. Gentry SE, Segev DL. Paired Exchange Programs for Living Donors. In: Knechtle SJ, Marson LP, Morris PJ, eds. *Kidney Transplantation - Principles and Practice*. 8 edn. Elsevier, 2019: 367-70.
82. Stepkowski SM, Mierzejewska B, Fumo D, et al. The 6-year clinical outcomes for patients registered in a multiregional United States Kidney Paired Donation program - a retrospective study. *Transpl Int*. 2019; **32**: 839-53.
83. Tietjen A, Hays R, McNatt G, et al. Billing for living kidney donor care: Balancing cost recovery, regulatory compliance, and minimized donor burden. *Curr Transplant Rep*. 2019; **6**: 155-66.
84. Valentín MO, Garcia M, Costa AN, et al. International cooperation for kidney exchange success. *Transplantation*. 2019; **103**: e180-e1.

85. Furian L, Nicolo A, Di Bella C, Cardillo M, Cozzi E, Rigotti P. Kidney exchange strategies: new aspects and applications with a focus on deceased donor-initiated chains. *Transpl Int*. 2020; **33**: 1177-84.
86. Kher V, Jha PK. Paired kidney exchange transplantation - pushing the boundaries. *Transpl Int*. 2020; **33**: 975-84.
87. Leeser DB, Thomas AG, Shaffer AA, et al. Patient and Kidney Allograft Survival with National Kidney Paired Donation. *Clin J Am Soc Nephrol*. 2020; **15**: 228-37.
88. McGregor TB, Sener A, Yetzer K, Gillrie C, Paraskevas S. The impact of COVID-19 on the Canadian Kidney Paired Donation program: an opportunity for universal implementation of kidney shipping. *Can J Surg*. 2020; **63**: E451-E3.
89. Nassiri N, Kwan L, Bolagani A, et al. The "oldest and coldest" shipped living donor kidneys transplanted through kidney paired donation. *Am J Transplant*. 2020; **20**: 137-44.
90. Shukhman E, Hunt J, LaPointe-Rudow D. Evaluation and care of international living kidney donor candidates: Strategies for addressing common considerations and challenges. *Clin Transplant*. 2020; **34**: e13792.
91. Syed B, Augustine JJ. The National Kidney Registry: time to buy in? *Clin J Am Soc Nephrol*. 2020; **15**: 168-70.
92. Verbesey J, Thomas AG, Ronin M, et al. Early graft losses in paired kidney exchange: Experience from 10 years of the National Kidney Registry. *Am J Transplant*. 2020; **20**: 1393-401.
93. Fortin MC, Gill J, Allard J, Ballesteros Gallego F, Gill J. Compatible Donor and Recipient Pairs' Perspectives on Participation in Kidney Paired Donation Programs: A Mixed-Methods Study. *Can J Kidney Health Dis*. 2021; **8**.
94. Hosseinzadeh A, Najafi M, Cheungpasitporn W, Thongprayoon C, Fathi M. Equity or Equality? Which Approach Brings More Satisfaction in a Kidney-Exchange Chain? *J Pers Med*. 2021; **11**: 1383.
95. Maghen A, Mendoza G, Vargas GB, et al. How Can We Help Alleviate the Financial Concerns of Non-Directed (Altruistic) Living Kidney Donors? *Prog Transplant*. 2021; **31**: 19-26.
96. van de Laar SC, Robb ML, Hogg R, Burnapp L, Papalois VE, Dor F. The Impact of Cold Ischaemia Time on Outcomes of Living Donor Kidney Transplantation in the UK Living Kidney Sharing Scheme. *Ann Surg*. 2021; **274**: 859-65.
97. Chipman V, Cooper M, Thomas AG, et al. Motivations and outcomes of compatible living donor-recipient pairs in paired exchange. *Am J Transplant*. 2022; **22**: 266-73.
98. Ong SC, Kumar V. *Kidney Paired Exchange: a step too far or a winning hand?* SciELO Brasil, 2022.
99. van de Laar SC, Lafranca JA, Minnee RC, Papalois V, Dor FJMF. The Impact of Cold Ischaemia Time on Outcomes of Living Donor Kidney Transplantation: A Systematic Review and Meta-Analysis. *J Clin Med*. 2022; **11**: 1620.
100. Kute VB, Fleetwood VA, Chauhan S, et al. Kidney paired donation in developing countries: A global perspective. *Curr Transplant Rep*. 2023; **10**: 117-25.
101. Duus Weinreich I, Andersson T, Birna Andrésdóttir M, et al. Scandiatransplant Exchange Program (STEP): Development and Results From an International Kidney Exchange Program. *Transplant Direct*. 2023; **9**: e1549.
102. Böhmig GA, Müller-Sacherer T. Kidney Paired Donation—European Transnational Experience in Adults and Opportunities for Pediatric Kidney Transplantation. *Pediatr Transplant*. 2024; **28**: e14840.
103. Francisco JT, Carvalho R, Freitas J. International Kidney Paired Donation–The Experience of a Single Center. *Braz J Transplant*. 2024.
104. Tirtayasa PMW, Situmorang GR, Duarsa GWK, et al. Risk factors of delayed graft function following living donor kidney transplantation: A meta-analysis. *Transpl Immunol*. 2024; **86**: 102094.
105. Verbesey J, Thomas AG, Waterman AD, et al. Unrecognized opportunities: The landscape of pediatric kidney-paired donation in the United States. *Pediatr Transplant*. 2024; **28**: e14657.
